# Machine learning model for predicting malnutrition risk in lung cancer patients after thoracoscopic resection: a multi-center study

**DOI:** 10.3389/fonc.2026.1727595

**Published:** 2026-02-09

**Authors:** Tianfeng Chen, Ruilan Pan, Ling Liang, Limei Xu, Mingyue Yang, Xiujuan Deng, Ping Wang

**Affiliations:** 1 School of Nursing, Guangdong Medical University, Dongguan, Guangdong, China; 2 Department of Thoracic Surgery, Affiliated Hospital of Guangdong Medical University, Zhanjiang, Guangdong, China; 3 Dongguan School of Clinical Medicine, Guangdong Medical University (Dongguan People’s Hospital), Dongguan, Guangdong, China

**Keywords:** lung cancer, machine learning, malnutrition, SHAP, thoracoscopic resection

## Abstract

**Background:**

Early detection of malnutrition is critical for timely intervention in lung cancer patients undergoing thoracoscopic resection. Existing black-box prediction models lack clinical interpretability, limiting trust and application. The present study was conducted to predict malnutrition risk by establishing an explainable machine learning (ML) model and evaluate the model performance across several sites, so as to develop a web-based application to aid clinical decision-making.

**Methods:**

A retrospective analysis was conducted on 1, 134 lung cancer patients who underwent thoracoscopic resection at Dongguan People’s Hospital between October 2021 and October 2024, consisting of a training set (n = 795) and a testing set (n = 339). Meanwhile, an external validation cohort (n=273) was prospectively enrolled at the Affiliated Hospital of Guangdong Medical University from March to June of 2025. Furthermore, univariate and multivariate analyses were employed to determine the individual risk variables for post-operative malnutrition. This study constructed eight ML models using Gradient Boosting Machine (GBM), Neural Network, Logistic Regression, Extreme Gradient Boosting (XGBoost), Random Forest, K-Nearest Neighbors (KNN), Adaptive Boosting (AdaBoost), and Support Vector Machine (SVM). The performance of the established models was assessed by decision curve analysis (DCA) and receiver operating characteristic (ROC) curves. Meanwhile, feature contributions and visualize model outputs were quantified using the SHapley Additive exPlanations (SHAP) method to enhance clinical interpretability. Consequently, a web-based risk calculator was created to assist in personalized forecasting.

**Results:**

Among 1, 407 total patients, post-operative malnutrition incidence was 11.3% (159/1, 407). Multivariate analysis identified seven independent risk factors: albumin (ALB), Nutritional Risk Screening 2002 score, age, intraoperative blood loss, total drainage volume, Basic Activities of Daily Living (BADL) score, and serum potassium (K). The XGBoost model outperformed others, with AUC 0.845 (95% CI: 0.771–0.919) in the testing set and 0.886 (95% CI: 0.841–0.932) in external validation. SHAP analysis clarified the relative importance of risk factors, improving interpretability.

**Conclusion:**

The XGBoost-based explainable ML model effectively predicts malnutrition risk in lung cancer patients after thoracoscopic resection. Integrating high predictive performance with interpretability, it supports clinical risk stratification and personalized nutritional interventions to improve post-operative outcomes. A publicly available web-based calculator facilitates easy clinical application.

## 1 Introduction

Malnutrition (1) refers to poor nutrition resulting from inadequate energy/nutrient intake or utilization disorders, characterized by abnormal body composition and impaired physiological function, and is an independent risk factor for cancer prognosis (2). Lung cancer remains the leading cause of cancer-related death worldwide (with approximately 2.5 million new cases and 1.8 million deaths annually); China, a country with a high incidence of lung cancer, recorded over 1.06 million new cases and 730, 000 deaths in 2022 (3, 4). The challenges are exacerbated by China’s large population, high smoking prevalence, and industrialization. Advances in diagnostics (5, 6) mean that more early-stage patients can undergo surgery. Video-assisted thoracoscopic resection has become the primary treatment modality due to fewer complications and faster recovery (7, 8). However, surgical stress induces hypermetabolism, activates immune-endocrine-metabolic cascades, and superimposes gastrointestinal dysfunction, leading to negative nitrogen balance and malnutrition (9). Postoperative malnutrition affects 17.5% - 54.2% of lung cancer patients (10, 11), prolonging mechanical ventilation duration, increasing complication risk, extending the length of stay in the hospital, and elevating mortality, while impairing quality of life and increasing family financial burdens (12, 13). Thus, identifying high-risk individuals is crucial for optimizing clinical decision-making.

Existing malnutrition risk prediction models exhibit several key limitations (14, 15): they predominantly employ traditional modeling approaches, rely on single-center datasets, lack external validation, and demonstrate suboptimal predictive performance. Critically, no dedicated models exist for thoracoscopic lung cancer patients, leaving a significant clinical gap for precise screening. Advances in artificial intelligence have enabled machine learning (ML) models to exhibit robust data processing and predictive capabilities, with great application potential in healthcare (16). Unlike traditional statistical models, explainable ML (e.g., SHapley Additive exPlanations (SHAP)) efficiently processes complex clinical data, quantifies each feature’s predictive importance, and converts “black box” decision-making into clinician-comprehensible logic, addressing the core limitation of traditional models in guiding clinical intervention priorities (17).

This study aimed to develop an interpretable ML model for predicting postoperative malnutrition in lung cancer patients undergoing thoracoscopic surgery. Distinct from existing malnutrition risk models and previous ML-based nutritional prediction studies, our work innovatively integrates a lung cancer-specific population undergoing thoracoscopic surgery with SHAP and prospective external validation, addressing the lack of dedicated and interpretable predictive tools for this specific cohort. We developed and validated eight ML models, selected the optimal model, quantified feature contributions via SHAP, and created a user-friendly online calculator. This work is expected to guide clinicians in prioritizing interventions and improving postoperative care quality.

## 2 Materials and methods

### 2.1 Design and participants

This study utilized a mixed design with simple random sampling applied for participant selection, incorporating both retrospective and prospective data. Retrospectively, this study collected data from lung cancer patients who had thoracoscopic resection at the Department of Thoracic Surgery, Dongguan People’s Hospital, Guangdong Province, from October 2021 to October 2024. These patients were randomly assigned to a training set and a testing set at a 7:3 ratio. Meanwhile, patients undergoing VATS for lung cancer at the Affiliated Hospital of Guangdong Medical University between March and June 2025 were sourced to get the prospective data and establish an external validation set. Class imbalance was not addressed during the data splitting process. Standardized perioperative management protocols were implemented at both centers, including routine preoperative education, standardized analgesia schemes, and unified postoperative dietary guidance, ensuring consistency in patient care across the study sites.

#### 2.1.1 Inclusion criteria

1. Participants aged ≥18 years;
2. Lung cancer patients who underwent thoracoscopic resection with pathologically confirmed lung cancer (18);
3. Patients with complete clinical and laboratory data available;
4. Participants with signed informed consent provided by themselves or their family members.

#### 2.1.2 Exclusion criteria

1. Patients with concurrent primary malignancies;
2. Patients with preoperative malnutrition, diagnosed per the 2019 Global Leadership Initiative on Malnutrition (GLIM) criteria (19);
3. Patients receiving postoperative radiotherapy, chemotherapy, neoadjuvant therapy, or targeted therapy;
4. Patients conversed to open thoracotomy during surgery;

#### 2.1.3 Sample size calculation

**Figure 1:**
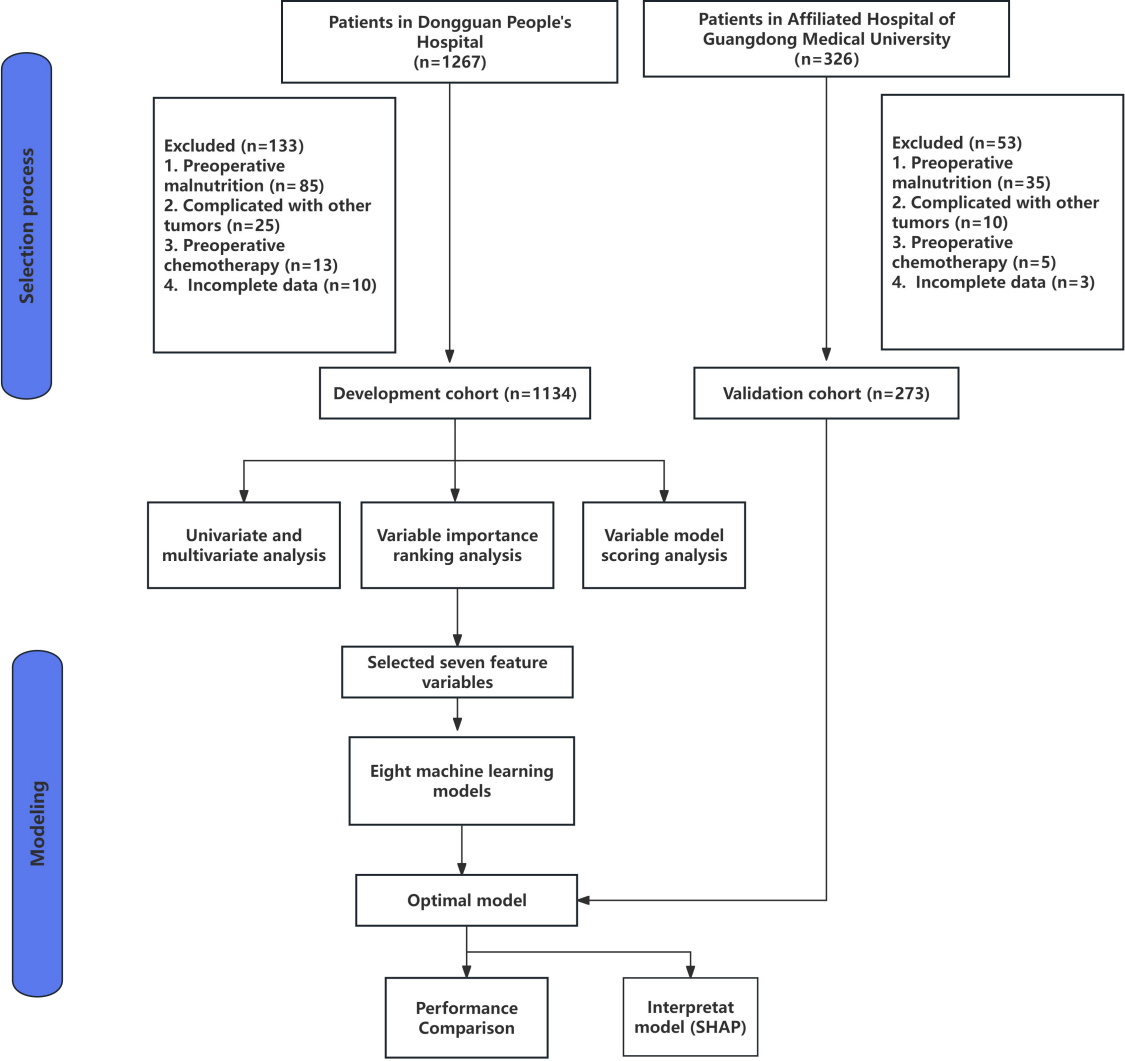
Flow diagram of patient selection workflow and model development methodology.

In our study, the minimum sample size was determined using the following equation: n=(Z_α/2_)^2^P(1-P)/δ^2^, where Z_α/2_ = 1.96 [95% confidence interval (95% CI), P = 0.273 (malnutrition incidence, referenced from Nakyeyune et al. (20)), and δ=3% (tolerance error). The required sample size was 942 given a 10% attrition rate. Finally, a total of 1, 407 eligible patients were included, including 795 cases in the retrospective training set, 339 cases in the retrospective testing set, and 273 cases in the prospective external validation set. **Figure 1** illustrates the detailed process of patient enrollment and dataset partitioning.

#### 2.1.4 Ethics approval

The study was officially approved by the Ethics Committee of Dongguan People’s Hospital, Guangdong Province (Approval No.: KYKT2025-011). It was also filed with the Ethics Committee of the Affiliated Hospital of Guangdong Medical University. With written informed consent provided by each patient or family member, this study was conducted strictly in accordance with the Declaration of Helsinki.

### 2.2 Malnutrition screening and diagnostic criteria

#### 2.2.1 Nutritional risk screening 2002 (NRS2002)

This study utilized the NRS2002 tool (21) to evaluate the nutritional risk of each patient. The tool comprises three components, with a total score ranging from 0 to 7 points. The scoring criteria are as follows:

**Table:** 

Scoring Item	Scoring Criteria	Score
A Nutritional status (0–3 points)	No weight loss in the past 3 months, and normal food intake.	0 point
	Weight loss in the past 3 months: >5%; or food intake reduction in the past week: 25%–50%.	1 point
	Weight loss in the past 3 months: 5%–10%; or food intake reduction in the past week: 50%–75%.	2 points
	Weight loss in the past 3 months: >10%; or food intake reduction in the past week: >75%; or BMI <18.5 kg/m² with impaired general condition	3 points
B Disease severity (0–3 points)	Normal nutritional requirements (e.g., elective surgery without complications).	0 point
	Slight increase in nutritional requirements (e.g., stable chronic diseases: liver cirrhosis, and chronic obstructive pulmonary disease).	1 point
	Moderate increase in nutritional requirements (e.g., major surgery, and acute exacerbation of chronic diseases: hip fracture, and stroke).	2 points
	Severe increase in nutritional requirements (e.g., sepsis, multiple organ failure, and severe trauma)	3 points
C Age (0–1 point)	<70 years.	0 point
	≥70 years.	1 point

Total Score = A + B + C.

Total Score ≥3 points: With nutritional risk, requiring further evaluation for the diagnosis of malnutrition.

Total Score <3 points: Without nutritional risk, with the recommendation of regular monitoring.

#### 2.2.2 Malnutrition diagnosis (GLIM consensus, 2019)

**Figure 2:**
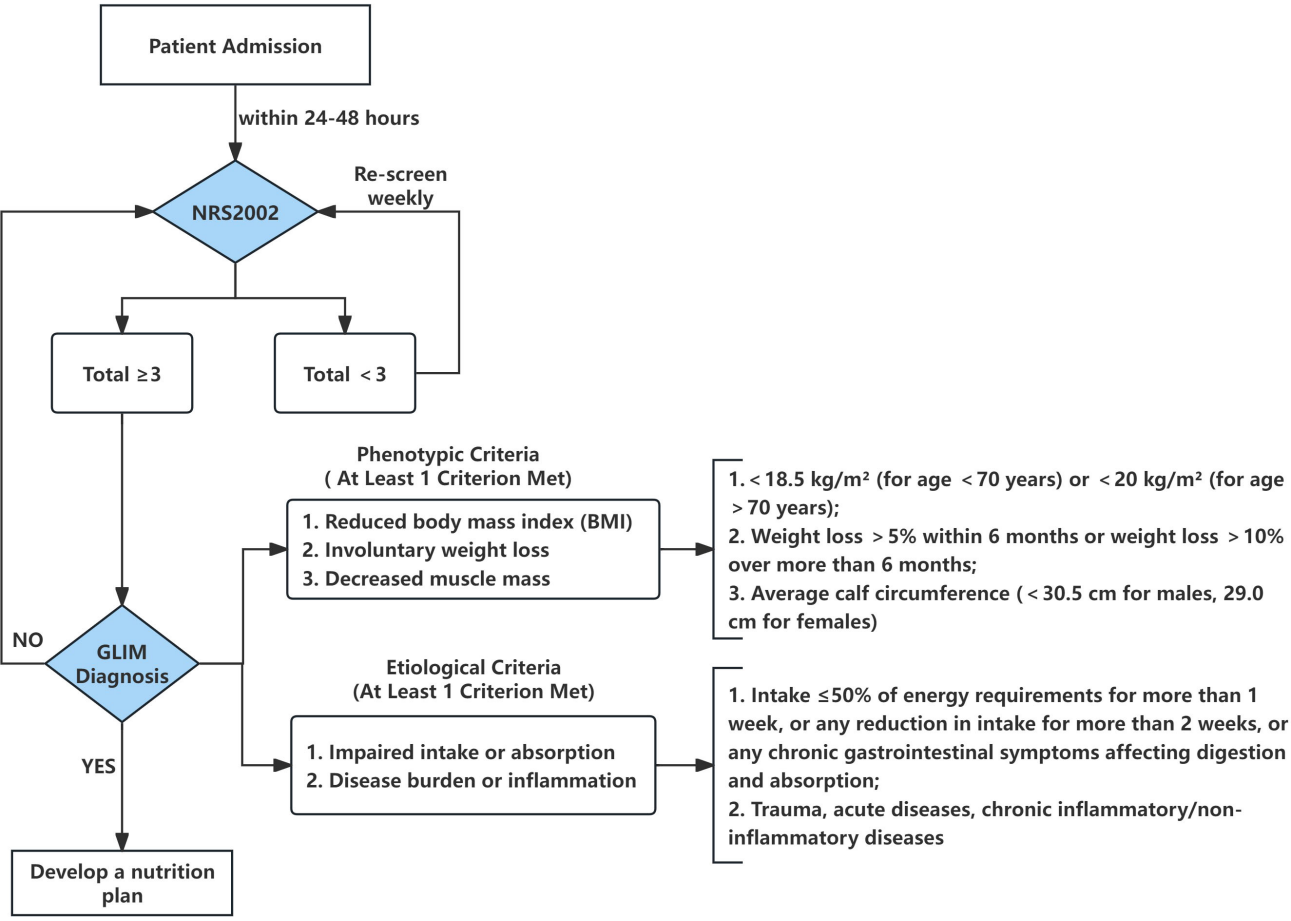
Flowchart of two-step malnutrition assessment: NRS2002 screening and GLIM diagnostic criteria (2019 consensus).

In this study, the diagnosis of malnutrition was achieved according to the two-step method suggested by the Global Leadership Initiative on the GLIM consensus (2019) (19). It involved the first step of confirming NRS2002 results, and the second step of combining one phenotypic criterion with one etiological criterion for the definitive diagnosis. Patients were continuously assessed until discharge, with the occurrence of malnutrition as the endpoint event. For clarity, the specific phenotypic and etiological criteria as well as decision pathway are detailed in **Figure 2**. Postoperative nutritional assessments were stratified by clinical status: weekly full NRS2002 screening was conducted for patients with stable conditions, while daily assessments were implemented for those with clinical changes (e.g., reduced food intake, postoperative complications, or abnormal laboratory results). A mandatory final nutritional assessment was performed for all patients before discharge. The endpoint of postoperative malnutrition was defined as the first confirmed diagnosis of malnutrition via the GLIM criteria during hospitalization; patients without a malnutrition diagnosis by discharge were classified into the non-malnutrition group.

### 2.3 Predictor variables

Based on literature review, group discussions, and clinical practice experience, this study collected eligible data using a self-designed “Clinical Data Survey Form for Patients After Thoracoscopic Lung Cancer Resection”. The survey covered the following three aspects:

#### 2.3.1 Demographic and baseline data

Specifically, the demographic and baseline data included patients’ name; gender; age; education level; smoking index (years smoked × cigarettes smoked per day); underlying conditions (e.g. chronic obstructive pulmonary disease, hypertension, diabetes mellitus, and tuberculosis); anthropometric measurements (e.g. height, weight, body mass index [BMI = weight/height²] and calf circumference at 10 cm below the patella); functional status assessment [e.g. Barthel Index for activities of daily living (ADL)] (22), with better functional status determined in case of higher scores, 100 points totally); and NRS2002 score.

#### 2.3.2 Disease- and surgery-related clinical data

Disease- and surgery-related clinical data encompassed tumour characteristics [pathological stage based on the 8th edition of the AJCC lung cancer staging system (18), tumour type (e.g., adenocarcinoma, squamous cell carcinoma, small cell carcinoma, etc.), and the number of lymph nodes dissected]; surgical data [surgical approach (e.g., lobectomy, segmentectomy, pneumonectomy), anaesthesia duration, and intraoperative blood loss]; total chest tube drainage volume; pain score (Numeric Rating Scale, 0–10 points) (23); complications (24); and constipation status (<1 bowel movement within 72 h postoperatively).

#### 2.3.3 Laboratory parameters

The following parameters were tested after the collection of fasting venous blood samples from the included patients within 24 h preoperatively.

- Biochemical markers: Total protein (TP), albumin (ALB), globulin (GLB), total cholesterol (TCH), blood glucose (GLU), and lactate dehydrogenase (LDH);
- Complete blood count parameters: Hemoglobin (Hb), white blood cell count (WBC), lymphocyte count (LYM), neutrophil count (NEUT), and platelet count (PLT);
- Electrolytes: Serum potassium (K), sodium (Na), chloride (Cl), and calcium (Ca).

### 2.4 Data collection and quality control

#### 2.4.1 Training and testing dataset collection

By sourcing the electronic medical record system and nursing records within the hospital, the demographic and sociological data, disease-related data, and laboratory test parameters of the enrolled patients were collected and organized by two nursing staff members from the Department of Thoracic Surgery who had undergone standardized training and passed the assessment. Among these, demographic and sociological data, as well as laboratory test-related data, were selected from the testing results of patients within 24 h of admission. Disease-related data included intraoperative and postoperative outcomes. According to the GLIM criteria for malnutrition assessment, patients were grouped into malnutrition and non-malnutrition categories.

#### 2.4.2 External validation dataset collection

Two researchers were responsible for follow-up assessments of patients after admission, using the NRS2002 and GLIM to evaluate patients until discharge. All other methods for data collection were consistent with those of the training and testing sets.

#### 2.4.3 Data quality control

A double-entry verification was performed using EpiData after the entry of all data into Excel by two researchers independently. Discrepancies were corrected by consulting the original records.

### 2.5 Statistical analyses

Data cleaning and database construction were performed using Excel. Statistical analysis and model development were carried out with the SPSS 26 and R 4.4.2 software packages (e.g., caret, pROC, Shapviz, and Shiny). Counting data were expressed as frequencies (n) and percentages (%), with inter-group comparisons performed using Fisher’s exact test or the chi-squared test. Meanwhile, measurement data distributed normally were expressed as mean ± standard deviation, and compared between groups by an independent samples t-test. Non-normally distributed measurement data in the form of median (interquartile range) [M (P_25_, P_75_)] were subjected to inter-group comparisons utilizing the Mann–Whitney U test. P < 0.05 denotes statistically significant differences, and α = 0.05 was chosen as the significance level. To verify the robustness of the selected features, Least Absolute Shrinkage and Selection Operator (LASSO) regression was performed. Continuous features were standardized via z-score normalization (R’s scale function) for model training stability. Hyperparameters were optimized by 10-fold internal cross-validation combined with GridSearchCV (caret package); optimal settings (selected by maximizing validation AUC) are detailed in **Supplementary Table**. Furthermore, calibration curves were plotted to assess the agreement between the predicted probabilities and the observed frequencies. To validate the model’s stability across different populations, stratified analysis was conducted in the external validation cohort based on age (< 65 vs. ≥ 65 years) and NRS2002 scores (< 3 vs. ≥ 3).

### 2.6 Development and evaluation of ML prediction models

(1) The training dataset was subjected to univariate and multivariate regression analyses. Logistic regression was applied to variables with statistical significance from the univariate analysis to identify independent factors affecting malnutrition in lung cancer patients after thoracoscopic resection. (2) Eight ML models were developed using Random Forest, Logistic Regression, Gradient Boosting Machine (GBM), Neural Network, XGBoost, K-Nearest Neighbors (KNN), Adaptive Boosting (AdaBoost), and Support Vector Machine (SVM). Then, the predictive performance of the constructed models was assessed based on ROC curves, AUC, sensitivity, specificity, precision, and F1-score. The model with the best performance was selected for further analysis. (3) Model validation. Internal validation and independent external validation were performed using 10-fold cross-validation to assess the robustness of the proposed model (25). (4) Model interpretation. Variable importance was assessed using the SHAP method. Finally, based on the optimal model, an interactive web calculator was developed using ShinyApps to facilitate clinical translation.

## 3 Results

### 3.1 Baseline characteristics

The study cohort consisted of 1, 407 suitable patients on the basis of strict inclusion and exclusion criteria. Of these, 1, 134 cases, dividing into a training set (n=795) and a testing set (n=339), were sourced from Dongguan People’s Hospital, Guangdong Province. The independent external validation set consisted of additional 273 cases from the Affiliated Hospital of Guangdong Medical University in Guangdong Province. As shown in **Table 1**, males accounted for 39.6% of the training set, 39.2% of the testing set, and 46.9% of the external validation set. The malnutrition rate was 10.94%, 10.62%, and 13.2% (slightly higher) in the training set, the testing set, and the external validation set, respectively. Compared to the training and testing cohorts, the external validation cohort had significantly higher mean age (56.96 ± 11.75 year and 57.78 ± 11.50 years vs. 61.20 ± 10.50 years, P < 0.001). The external validation cohort exhibited a higher proportion of patients with an NRS2002 score ≥ 3 compared to the training and testing cohorts (23.4% vs. 12.1% and 12.7%; P < 0.001).

**Table 1:** Baseline characteristics of patients with lung cancer in the training, testing, and external validation cohorts.

Name	Training set	Testing set	Validation set	p
n	795	339	273	
Gender = Male (%)	315 (39.6)	133 (39.2)	128 (46.9)	0.083
Educational Level (%)				0.001
Lower secondary education	290 (36.5)	126 (37.2)	74 (27.1)	
Tertiary education and above	77 (9.7)	43 (12.7)	18 (6.6)	
Upper secondary education	428 (53.8)	170 (50.1)	181 (66.3)	
NRS2002 (%)				<0.001
1	593 (74.6)	242 (71.4)	194 (71.1)	
2	106 (13.3)	54 (15.9)	15 (5.5)	
3	89 (11.2)	41 (12.1)	62 (22.7)	
4	7 (0.9)	2 (0.6)	0 (0.0)	
5	0 (0.0)	0 (0.0)	2 (0.7)	
Surgical Approach (%)				<0.001
Pulmonary Lobectomy	50 (6.3)	18 (5.3)	35 (12.8)	
Pulmonary Segmentectomy	342 (43.1)	142 (41.9)	66 (24.2)	
Pulmonary Wedge Resection	111 (14.0)	52 (15.3)	67 (24.5)	
Radical Lung Cancer Resection	291 (36.6)	127 (37.5)	105 (38.5)	
Number of Dissected Lymph Nodes (%)				<0.001
≤3	197 (24.8)	91 (26.8)	126 (46.2)	
≥8	86 (10.8)	33 (9.7)	5 (1.8)	
4~7	512 (64.4)	215 (63.4)	142 (52.0)	
Pathological Staging (%)				<0.001
Stage 0	87 (10.9)	28 (8.3)	30 (11.0)	
Stage 1	648 (81.5)	289 (85.3)	204 (74.7)	
Stage 2	37 (4.7)	17 (5.0)	12 (4.4)	
Stage 3	14 (1.8)	4 (1.2)	21 (7.7)	
Stage 4	9 (1.1)	1 (0.3)	6 (2.2)	
Tumour Type (%)				0.291
Adenocarcinoma	762 (95.8)	324 (95.6)	267 (97.8)	
Other Tumor Types	13 (1.6)	3 (0.9)	1 (0.4)	
Squamous Cell Carcinoma	20 (2.5)	12 (3.5)	5 (1.8)	
Pain (%)				<0.001
1	5 (0.6)	1 (0.3)	91 (33.3)	
2	467 (58.7)	190 (56.0)	151 (55.3)	
3	270 (34.0)	118 (34.8)	27 (9.9)	
4	43 (5.4)	20 (5.9)	3 (1.1)	
5	7 (0.9)	8 (2.4)	1 (0.4)	
6	3 (0.4)	2 (0.6)	0 (0.0)	
Constipation = Yes (%)	503 (63.3)	200 (59.0)	151 (55.3)	0.051
Complications = Yes (%)	87 (10.9)	35 (10.3)	32 (11.7)	0.859
Underlying Disease = Yes (%)	345 (43.4)	141 (41.6)	68 (24.9)	<0.001
Age (mean (SD))	56.96 (11.75)	57.78 (11.50)	61.20 (10.50)	<0.001
BMI (mean (SD))	23.69 (2.82)	23.71 (3.00)	22.78 (2.74)	<0.001
Calf Circumference (mean (SD))	33.43 (2.86)	33.69 (2.91)	32.22 (2.82)	<0.001
Anesthesia duration(mean (SD))	3.79 (1.07)	3.79 (1.04)	4.10 (1.57)	<0.001
Blood Loss (mean (SD))	41.60 (116.01)	31.56 (44.77)	28.70 (55.94)	0.073
Total Drainage Volume (mean (SD))	524.82 (439.74)	516.95 (427.02)	576.74 (648.26)	0.244
BADL (mean (SD))	99.57 (3.32)	99.40 (3.24)	99.29 (3.48)	0.436
Smoking_index (mean (SD))	105.07 (321.59)	73.78 (294.44)	62.86 (217.00)	0.07
WBC (mean (SD))	6.22 (1.79)	6.26 (1.91)	7.85 (3.08)	<0.001
NEUT (mean (SD))	3.82 (1.68)	3.83 (1.70)	5.51 (3.17)	<0.001
LYM (mean (SD))	1.81 (0.70)	1.80 (0.85)	1.62 (0.71)	0.001
HGB (mean (SD))	133.21 (28.57)	131.81 (25.08)	128.93 (15.86)	0.059
PLT (mean (SD))	230.75 (60.12)	230.93 (61.12)	238.74 (71.95)	0.173
LDH (mean (SD))	179.25 (37.55)	177.12 (33.29)	199.89 (47.35)	<0.001
TP (mean (SD))	68.61 (5.13)	68.38 (5.78)	68.41 (6.43)	0.767
ALB (mean (SD))	40.45 (3.31)	40.51 (3.72)	40.95 (3.60)	0.114
GLB (mean (SD))	28.05 (3.79)	27.81 (4.26)	27.49 (4.41)	0.134
GLU (mean (SD))	5.34 (1.29)	5.42 (1.40)	5.35 (1.15)	0.647
TCH (mean (SD))	4.90 (1.04)	4.83 (1.05)	4.90 (1.07)	0.535
K (mean (SD))	3.91 (0.32)	3.92 (0.33)	3.90 (0.36)	0.772
Na (mean (SD))	140.18 (2.47)	140.11 (2.47)	139.45 (2.53)	<0.001
Cl (mean (SD))	105.14 (2.64)	105.18 (2.67)	102.72 (5.72)	<0.001
Ca (mean (SD))	2.27 (0.11)	2.24 (0.12)	2.22 (0.12)	<0.001

WBC, White Blood Cell; NEUT, Neutrophil; LYM, Lymphocyte; HGB, Hemoglobin;

PLT, Platelet; LDH, Lactate Dehydrogenase; TP, Total Protein; ALB, Albumin;

GLB, Globulin; GLU, Glucose; TCH, Total Cholesterol.

### 3.2 Feature selection

In our study, malnutrition status (binary outcome variable defined by the GLIM criteria) was determined as the dependent variable. Thirty-four clinical features were included as independent variables. In univariate analysis (1 sample excluded), there were statistically significant differences in 16 variables between the malnutrition group and the non-malnutrition group (all P < 0.05). Furthermore, multivariate logistic regression analysis identified NRS2002, age, blood loss, total drainage volume, BADL, and serum K level as independent nutritional risk variables (OR > 1, P < 0.05), while ALB as an independent protective factor (OR < 1, P < 0.05) (**Table 2**).

**Table 2:** Univariate and multivariate evaluations of malnutrition-related factors.

Name	Desc	No (N = 707)	Yes (N = 87)	OR (Univariate)	OR (Multivariable)
Gender	Female	437 (61.8%)	42 (48.3%)		
	Male	270 (38.2%)	45 (51.7%)	1.73 (1.11-2.71, p=0.016)	1.01 (0.50-2.02, p=0.987)
Educational Level	Lower secondary education	256 (36.2%)	34 (39.1%)		
	Upper secondary education	381 (53.9%)	46 (52.9%)	0.91 (0.57-1.46, p=0.691)	
	Tertiary education and above	70 (9.9%)	7 (8%)	0.75 (0.32-1.77, p=0.515)	
NRS2002	1	559 (79.1%)	33 (37.9%)		
	2	81 (11.5%)	25 (28.7%)	5.23 (2.96-9.24, p<0.001)	2.31 (0.95-5.64, p=0.066)
	3	67 (9.5%)	22 (25.3%)	5.56 (3.06-10.09, p<0.001)	9.22 (3.54-23.99, p<0.001)
	4	0 (0%)	7 (8%)		
Surgical Approach	Pulmonary Wedge Resection	99 (14%)	12 (13.8%)		
	Pulmonary Segmentectomy	317 (44.8%)	25 (28.7%)	0.65 (0.32-1.34, p=0.245)	
	Pulmonary Lobectomy	45 (6.4%)	5 (5.7%)	0.92 (0.30-2.76, p=0.877)	
	Radical Lung Cancer Resection	246 (34.8%)	45 (51.7%)	1.51 (0.77-2.97, p=0.234)	
Number of Dissected Lymph Nodes	≤3	178 (25.2%)	19 (21.8%)		
	4~7	455 (64.4%)	56 (64.4%)	1.15 (0.67-2.00, p=0.611)	
	≥8	74 (10.5%)	12 (13.8%)	1.52 (0.70-3.29, p=0.288)	
Pathological Staging	Stage 0	82 (11.6%)	5 (5.7%)		
	Stage 1	572 (80.9%)	76 (87.4%)	2.18 (0.86-5.55, p=0.102)	
	Stage 2	32 (4.5%)	4 (4.6%)	2.05 (0.52-8.12, p=0.307)	
	Stage 3	12 (1.7%)	2 (2.3%)	2.73 (0.48-15.70, p=0.260)	
	Stage 4	9 (1.3%)	0 (0%)		
Tumour Type	Adenocarcinoma	682 (96.5%)	80 (92%)		
	Squamous Cell Carcinoma	14 (2%)	5 (5.7%)	3.04 (1.07-8.67, p=0.037)	0.51 (0.10-2.56, p=0.410)
	Other Tumor Types	11 (1.6%)	2 (2.3%)	1.55 (0.34-7.12, p=0.573)	3.25 (0.60-17.67, p=0.172)
Pain	1	4 (0.6%)	1 (1.1%)		
	2	425 (60.1%)	41 (47.1%)	0.39 (0.04-3.53, p=0.399)	
	3	236 (33.4%)	34 (39.1%)	0.58 (0.06-5.31, p=0.627)	
	4	35 (5%)	8 (9.2%)	0.91 (0.09-9.32, p=0.940)	
	5	4 (0.6%)	3 (3.4%)	3.00 (0.21-42.62, p=0.417)	
	6	3 (0.4%)	0 (0%)		
Constipation	No	266 (37.6%)	26 (29.9%)		
	Yes	441 (62.4%)	61 (70.1%)	1.42 (0.87-2.30, p=0.159)	
Complications	No	632 (89.4%)	75 (86.2%)		
	Yes	75 (10.6%)	12 (13.8%)	1.35 (0.70-2.59, p=0.371)	
Underlying Disease	No	409 (57.9%)	40 (46%)		
	Yes	298 (42.1%)	47 (54%)	1.61 (1.03-2.52, p=0.036)	0.97 (0.52-1.83, p=0.931)
Age	Mean ± SD	56.1 ± 11.5	64.1 ± 11.6	1.07 (1.05-1.10, p<0.001)	1.04 (1.00-1.08, p=0.029)
BMI	Mean ± SD	23.8 ± 2.7	23.0 ± 3.5	0.90 (0.83-0.98, p=0.021)	0.95 (0.81-1.12, p=0.561)
Calf Circumference	Mean ± SD	33.5 ± 2.8	32.7 ± 3.3	0.91 (0.84-0.98, p=0.015)	1.07 (0.93-1.23, p=0.332)
Anesthesia Time	Mean ± SD	3.8 ± 1.0	4.1 ± 1.5	1.27 (1.05-1.54, p=0.013)	0.78 (0.59-1.03, p=0.084)
Blood Loss	Mean ± SD	28.8 ± 30.8	145.5 ± 322.8	1.01 (1.00-1.01, p<0.001)	1.01 (1.00-1.01, p<0.001)
Total Drainage Volume	Mean ± SD	489.9 ± 337.1	811.0 ± 871.2	1.00 (1.00-1.00, p<0.001)	1.00 (1.00-1.00, p<0.001)
BADL	Mean ± SD	99.8 ± 1.3	97.5 ± 9.1	0.86 (0.79-0.94, p=0.001)	0.89 (0.81-0.98, p=0.015)
Smoking Index	Mean ± SD	95.0 ± 297.8	188.3 ± 467.8	1.00 (1.00-1.00, p=0.014)	1.00 (1.00-1.00, p=0.540)
WBC	Mean ± SD	6.2 ± 1.7	6.4 ± 2.5	1.05 (0.94-1.19, p=0.373)	
NEUT	Mean ± SD	3.8 ± 1.6	4.1 ± 2.4	1.11 (0.99-1.24, p=0.069)	
LYM	Mean ± SD	1.8 ± 0.7	1.6 ± 0.6	0.58 (0.38-0.88, p=0.011)	0.74 (0.43-1.28, p=0.283)
HGB	Mean ± SD	133.4 ± 27.9	132.2 ± 33.6	1.00 (0.99-1.01, p=0.725)	
PLT	Mean ± SD	230.9 ± 58.2	230.3 ± 74.1	1.00 (1.00-1.00, p=0.929)	
LDH	Mean ± SD	179.0 ± 36.5	182.0 ± 45.5	1.00 (1.00-1.01, p=0.480)	
TP	Mean ± SD	68.8 ± 5.1	66.9 ± 5.2	0.93 (0.89-0.97, p=0.001)	0.98 (0.91-1.05, p=0.540)
ALB	Mean ± SD	40.7 ± 3.1	38.7 ± 4.1	0.84 (0.78-0.90, p<0.001)	0.90 (0.80-1.00, p=0.046)
GLB	Mean ± SD	28.0 ± 3.7	28.1 ± 4.4	1.01 (0.95-1.07, p=0.816)	
GLU	Mean ± SD	5.3 ± 1.3	5.6 ± 1.3	1.14 (0.99-1.31, p=0.062)	
TCH	Mean ± SD	4.9 ± 1.0	4.7 ± 1.1	0.85 (0.68-1.07, p=0.160)	
K	Mean ± SD	3.9 ± 0.3	4.0 ± 0.3	3.52 (1.74-7.14, p<0.001)	4.07 (1.66-9.98, p=0.002)
Na	Mean ± SD	140.2 ± 2.5	140.2 ± 2.5	1.00 (0.91-1.09, p=0.935)	
Cl	Mean ± SD	105.1 ± 2.6	105.4 ± 3.2	1.04 (0.95-1.14, p=0.362)	
Ca	Mean ± SD	2.3 ± 0.1	2.3 ± 0.1	0.19 (0.03-1.32, p=0.094)	

**Figure 3:**
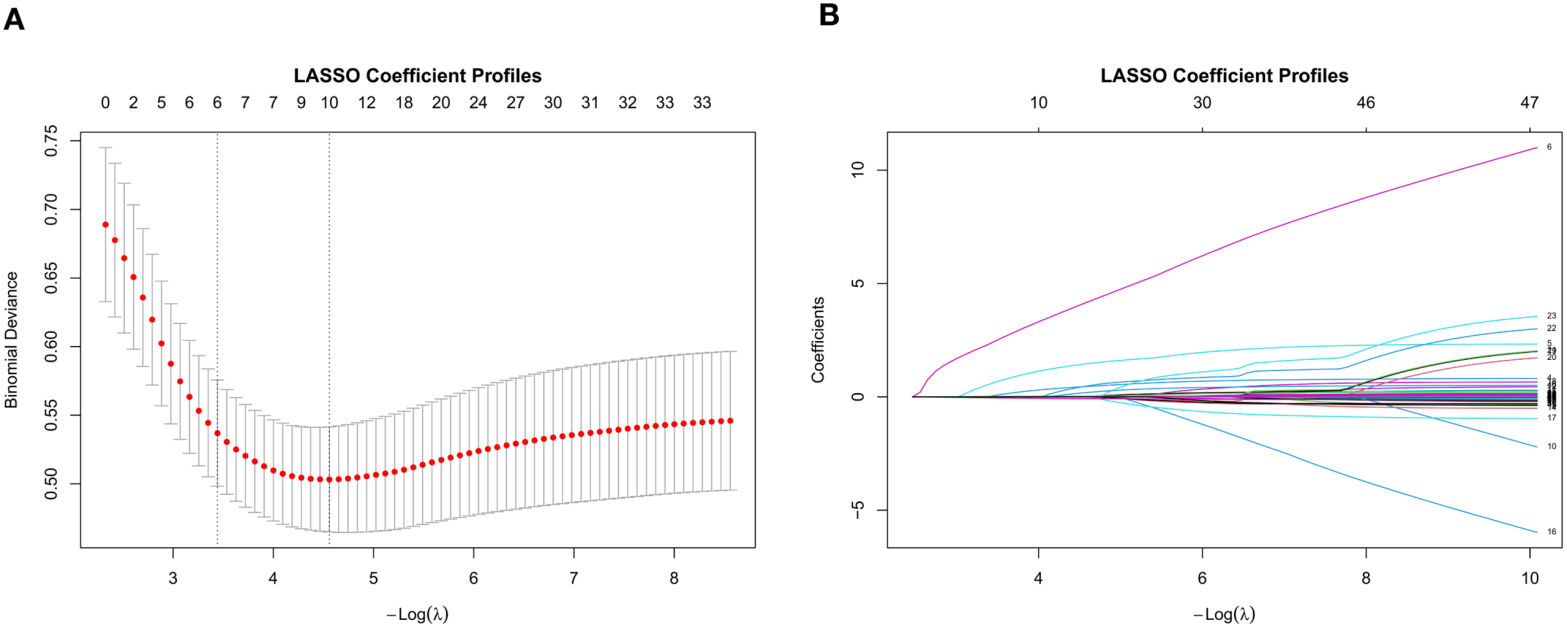
LASSO binary logistic regression for feature selection. **(A)** Binomial deviance curve of the LASSO model. The dashed line indicates the optimal λ value selected by 10-fold cross-validation. **(B)** Coefficient path of the LASSO model. Colored lines represent feature coefficients, with non-zero values at optimal λ indicating selected features.

The robustness of these seven features was further confirmed by LASSO regression analysis (**Figure 3**), which showed consistent results with the multivariate logistic regression.

### 3.3 Model construction and validation

Based on the 7 selected clinical features in multivariate analysis, eight typical machine learning methods were used to construct predictive models. Model fitting evaluations and stability screening were implemented to eliminate overfitted models, followed by a comprehensive assessment using calibration curve, ROC, DCA, AUC, sensitivity, specificity, precision, and F1-score.

**Figure 4:**
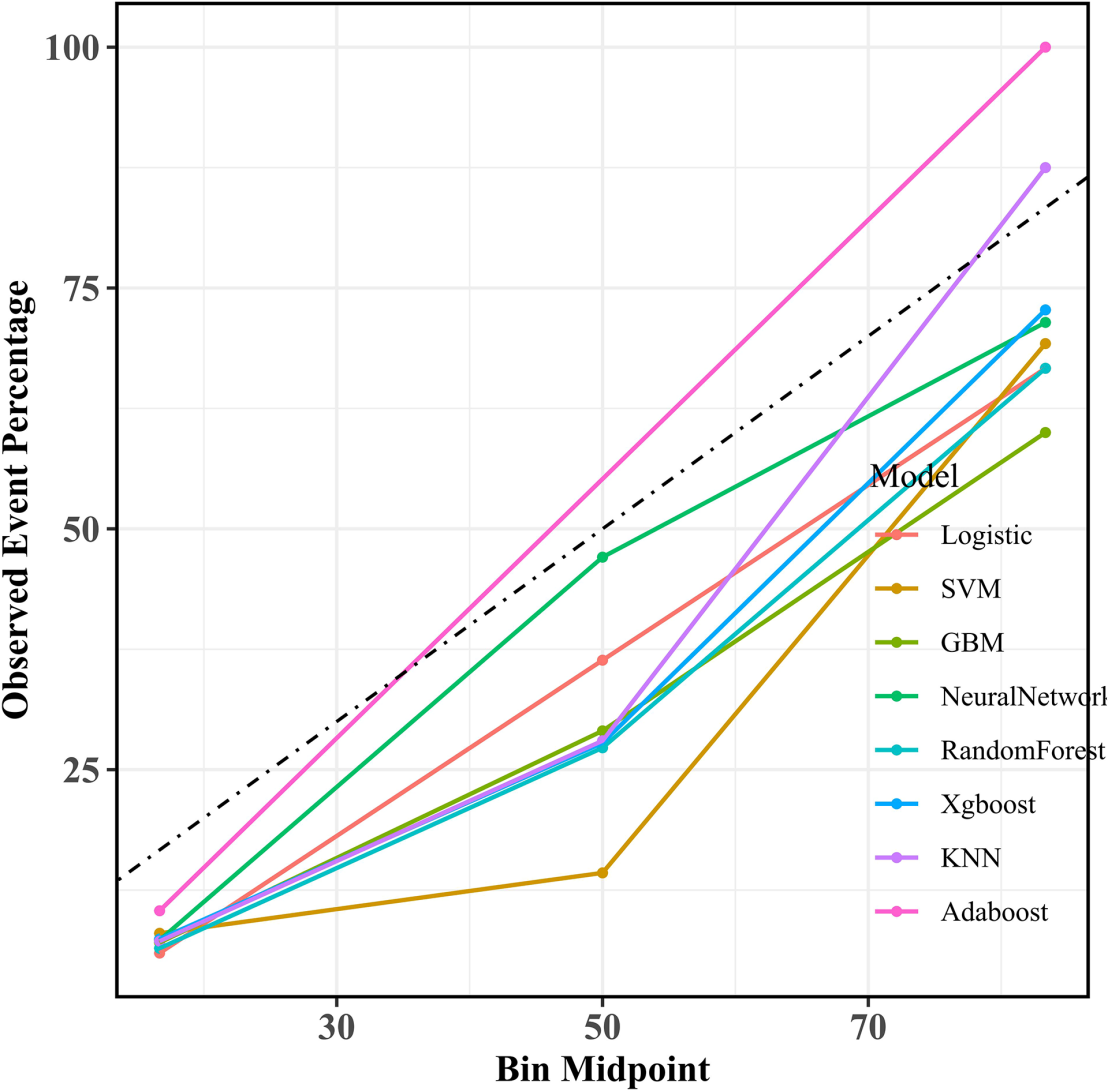
Calibration curves of multiple machine learning models on the testing set.

Calibration analysis demonstrated good agreement between the predicted risk and actual malnutrition incidence. As shown in **Figure 4**, the calibration curve of the XGBoost model aligned closely with the ideal diagonal line, indicating high reliability in risk estimation.

In the comparison of the AUC values, the RandomForest model achieved an AUC of 0.994 and 0.802 on the training and testing sets, respectively, with a difference of 0.192. Furthermore, the sensitivity of the testing set was obviously lower than that of the training set (72.2% vs. 98.9%), indicating overfitting. Therefore, it was ultimately not selected as the optimal model. Simultaneously, the RandomForest model was also subjected to analyses using methods such as pruning and increasing the sample size, but the results were unsatisfactory. Consequently, the optimal model was determined to be XGBoost, which showed excellent ability of generalization.

**Figure 5:**
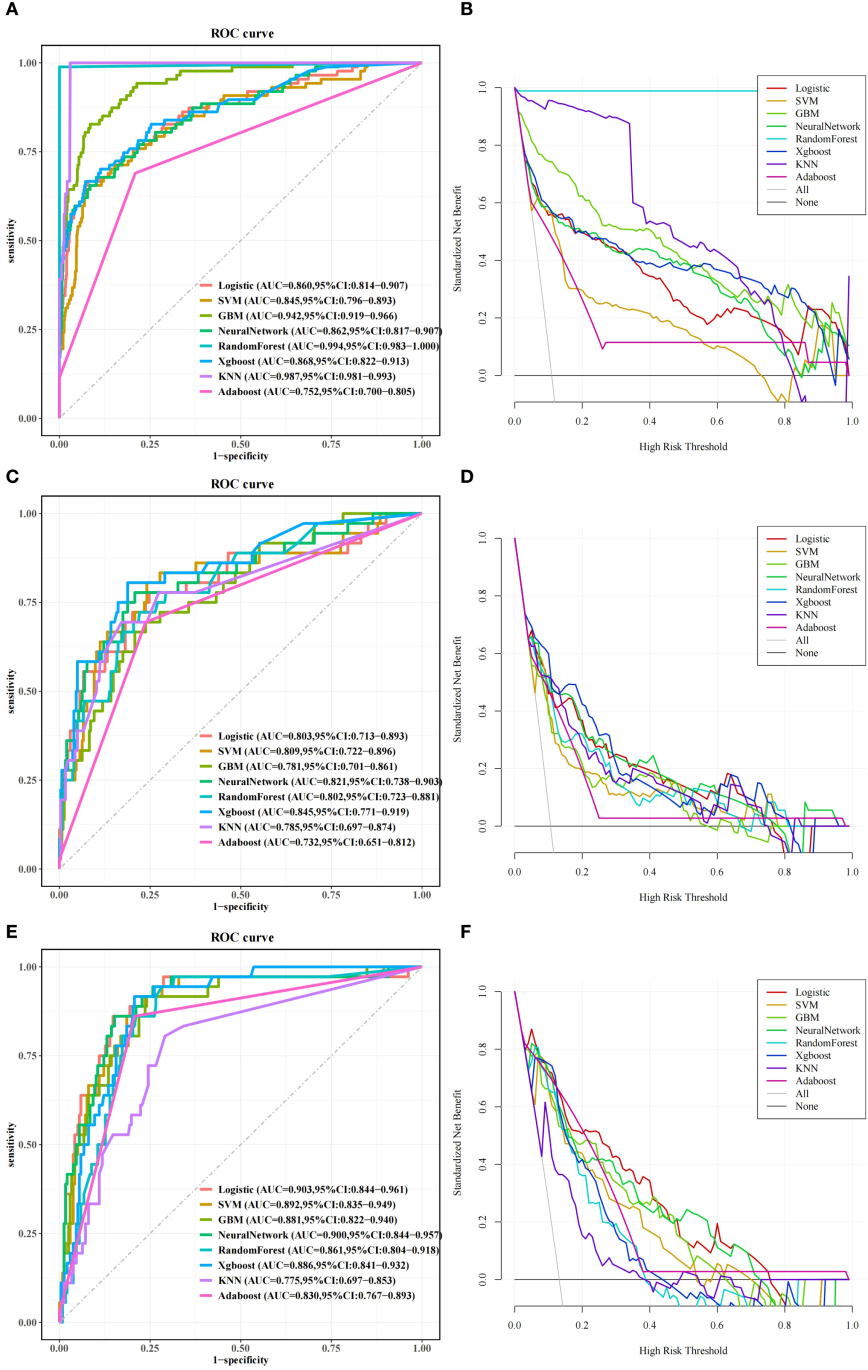
ROC and DCA curves across training, testing, and external validation sets. **(A)** ROC curves for the eight models in the training set. **(C)** ROC curves for the eight models in the testing set. **(E)** ROC curves for the eight models in the external validation set. **(B)** DCA for the eight models in the training set. **(D)** DCA for the eight models in the testing set. **(F)** The DCA of the eight models in the external validation set.

In the independent external validation cohort, the XGBoost model maintained stable performance (AUC = 0.886, 95% CI: 0.841–0.932), validating its robustness across different clinical settings and potential for translation in real-world practice (**Figure 5** and **Table 3**). Given the significant differences in baseline characteristics (older age and higher nutritional risk) between the training and external validation cohorts, a stratified analysis was further conducted to evaluate the model’s robustness in specific subgroups. The XGBoost model demonstrated excellent discriminative ability across age groups, with an AUC of 0.943 (95% CI: 0.902–0.984) for patients < 65 years and 0.822 (95% CI: 0.737–0.906) for those $\ge$ 65 years. Regarding nutritional status, the model performed well in the majority of patients with low-to-moderate risk (NRS2002 < 3; AUC: 0.829, 95% CI: 0.617–1.000). However, attenuated performance was observed in the high-risk subgroup (NRS2002 $\ge$ 3; AUC: 0.600, 95% CI: 0.460–0.740), likely reflecting the increased clinical complexity and limited sample size (n = 64) in this specific subset (**Table 4**).

**Table 3:** Comparative performance metrics of ML algorithms in predicting malnutrition risk in lung cancer patients undergoing thoracoscopic resection.

Model	AUC	Accuracy	Sensitivity	Specificity	Precision	F1
Training set						
Logistic	0.86	0.893	0.667	0.921	0.509	0.577
SVM	0.845	0.887	0.655	0.915	0.487	0.559
GBM	0.942	0.906	0.828	0.915	0.545	0.658
NeuralNetwork	0.862	0.874	0.678	0.898	0.45	0.541
RandomForest	0.994	0.999	0.989	1	1	0.994
Xgboost	0.868	0.899	0.667	0.928	0.532	0.592
KNN	0.987	0.974	1	0.97	0.806	0.892
Adaboost	0.752	0.78	0.69	0.791	0.288	0.407
Testing set						
Logistic	0.803	0.755	0.778	0.752	0.272	0.403
SVM	0.809	0.764	0.806	0.759	0.284	0.42
GBM	0.781	0.779	0.667	0.792	0.276	0.39
NeuralNetwork	0.821	0.791	0.778	0.792	0.308	0.441
RandomForest	0.802	0.773	0.722	0.779	0.28	0.403
Xgboost	0.845	0.811	0.806	0.812	0.337	0.475
KNN	0.785	0.814	0.694	0.828	0.325	0.442
Adaboost	0.732	0.755	0.694	0.762	0.258	0.376
Validation set						
Logistic	0.903	0.853	0.861	0.852	0.47	0.608
SVM	0.892	0.81	0.889	0.797	0.4	0.552
GBM	0.881	0.78	0.917	0.759	0.367	0.524
NeuralNetwork	0.900	0.85	0.861	0.848	0.463	0.602
RandomForest	0.861	0.747	0.944	0.717	0.337	0.496
Xgboost	0.886	0.81	0.917	0.793	0.402	0.559
KNN	0.775	0.722	0.806	0.709	0.296	0.433
Adaboost	0.830	0.802	0.861	0.793	0.388	0.534

**Table 4:** Stratified validation of model robustness: AUC across age and NRS2002 subgroups.

Subgroup	Sample size (N)	AUC	95% CI
Age			
< 65 years	156	0.943	0.902-0.984
≥ 65 years	117	0.822	0.737-0.906
NRS2002 Score			
< 3	209	0.829	0.617-1.000
≥ 3	64	0.600	0.460-0.740

### 3.4 SHAP analysis and model interpretability of the XGBoost ML model

**Figure 6:**
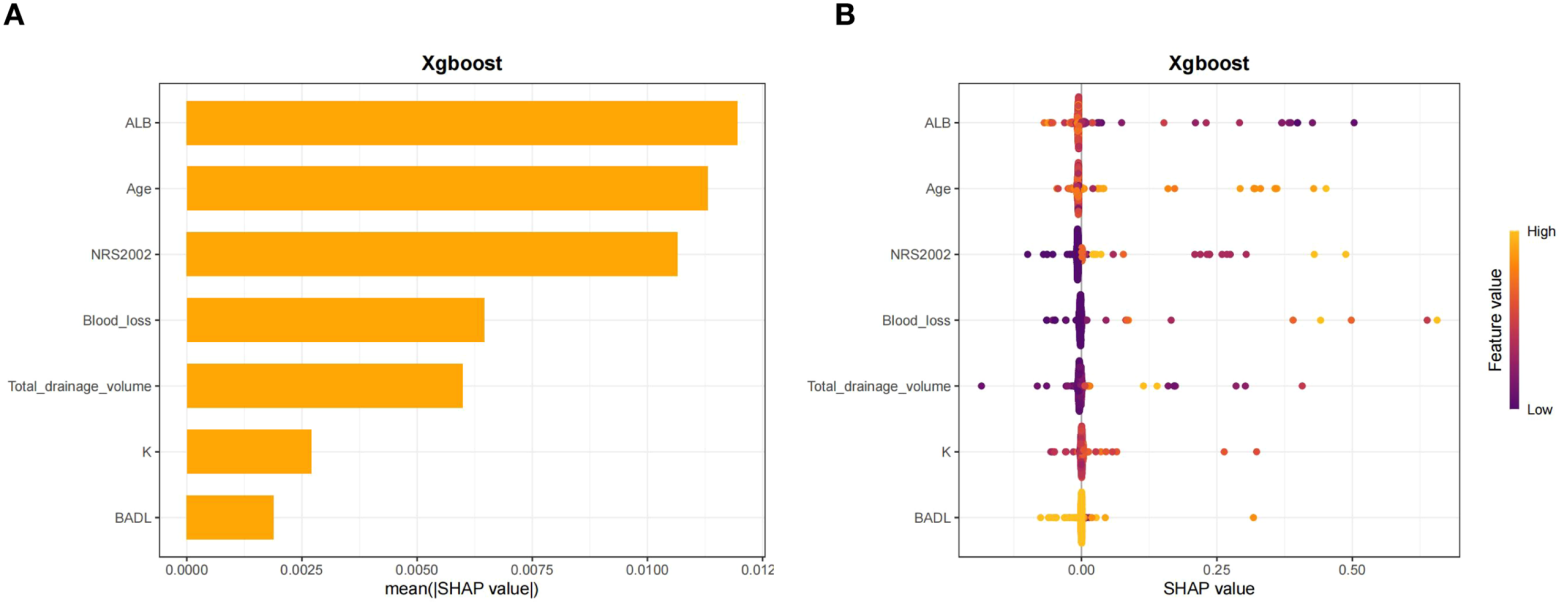
XGBoost model interpretability: **(A)** Feature Importance Ranking, and **(B)** SHAP Value Distribution.

The SHAP algorithm was used to measure the significance and contribution of each predictor variable. As depicted in **Figure 6A**, feature importance was ranked in descending order by average SHAP absolute value as follows: ALB > age > NRS2002 score > intraoperative blood loss > total thoracic drainage volume > K > BADL score. As a result, ALB was identified as the core variable with the strongest predictive capability in the model.

This study continued to identify the correlation between variables and prediction outcomes using the SHAP_XGBoost_importance_beeswarm plot (**Figure 6B**). Specifically, increased NRS2002 scores and advancing age were both positively correlated with SHAP values, indicating that both of which were independent risk factors for malnutrition. Conversely, elevated ALB level exhibited a negative correlation with the SHAP value, suggesting it acting as a protective factor against malnutrition, consistent with clinical nutritional assessment logic.

Based on the aforementioned XGBoost model, this study developed an online malnutrition risk assessment tool (https://chentianfeng630077.shinyapps.io/make_web). Construction of this tool would support real-time input of key clinical indicators and personalized risk prediction, providing a convenient approach for rapid clinical screening. The optimal threshold of this model was identified as 0.4086 for predicting malnutrition risk.

**Figure 7:**
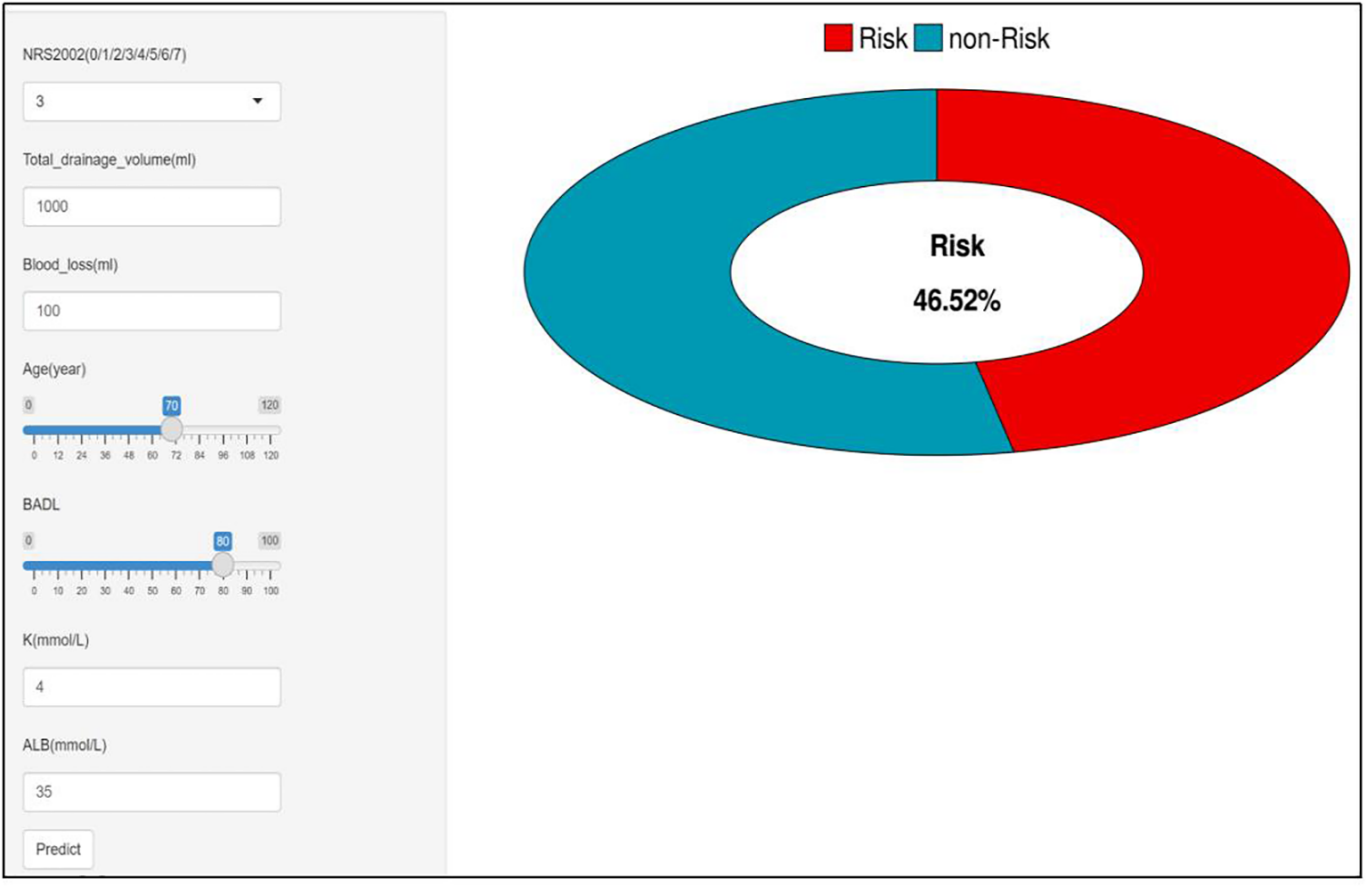
Computational results of online website for postoperative malnutrition in lung cancer patients undergoing thoracoscopic resection.

Eventually, in order to validate the practical efficacy of the tool, this study employed simple random sampling to select a lung cancer inpatient with characteristics below: Age: 70 years; ALB: 35 g/L; NRS2002 score: 3 points; intraoperative blood loss: 100 mL; total thoracic drainage volume: 1, 000 mL; BADL score: 80 points; and K: 4 mmol/L. A postoperative malnutrition risk of 46.52% for this patient (> 0.4086) was yielded when inputting these variables into the XGBoost-based online postoperative malnutrition risk prediction model (**Figure 7**). Via follow-up, the patient was identified to develop postoperative malnutrition, validating the consistency between prediction result of this model and clinical outcomes.

## 4 Discussion

### 4.1 Favorable performance of the model for accurately predicting malnutrition risk in lung cancer patients undergoing thoracoscopic resection

This study analyzed the medical data of patients with lung cancer who underwent thoracoscopic surgery at two medical centers. We employed multivariate logistic regression analysis to determine key feature variables, followed by the construction and evaluation of prediction models by using eight mainstream ML prediction models. Comprehensive evaluation of the constructed model performance revealed that the XGBoost model exhibited the optimal performance and significantly outperformed other models, with superior discrimination and stability across the training set (AUC = 0.868), testing set (AUC = 0.845), and external validation set (AUC = 0.886). SHAP analysis identified seven core features, including ALB, Age, NRS2002, blood loss, total drainage volume, K, and BADL. Their influence was ranked as follows: ALB > age > NRS2002 > blood loss > total drainage volume > K > BADL.

Jiang Y et al. (26) developed three risk prediction models for malignant pleural effusion in lung cancer patients, with result visualization via nomogram, yet without the internal validation. In contrast, our model exhibited advantages of improving prediction accuracy as well as enhancing model transparency and clinical guidance as it integrated the SHAP method to comprehensively evaluate multiple factors. Similarly, Li et al. (27) constructed a prediction model for postoperative exercise phobia, with an AUC of 0.893. But their study primarily incorporated subjective variables, without external validation, and failed to develop a web-based assessment tool to support its clinical applicability.

Leveraging the advantages outlined above, our XGBoost model demonstrated broad applicability. Notably, our stratified analysis confirmed the model’s robustness in elderly patients (Age ≥ 65), with an AUC exceeding 0.80, which effectively addresses concerns regarding model applicability in the aging lung cancer population. However, we observed reduced predictive accuracy in patients with high pre-existing nutritional risk (NRS2002 ≥ 3). This ‘ceiling effect’ suggests that distinguishing outcomes within an already high-risk cohort is inherently challenging using standard clinical variables. For this specific subgroup, malnutrition may be driven by more complex metabolic or immune factors not fully captured in the current model, warranting closer clinical monitoring, serving as a complement to the model’s risk stratification. Clinically, healthcare providers can input the seven key indicators identified in our study into the online prediction tool to generate real-time postoperative malnutrition risk scores. Based on clinical practice needs, it is recommended to carry out high-risk classification for patients with a risk of ≥40.86%, triggering daily nutritional assessments and individualized interventions subsequently. Simultaneously, those with a risk of <40.86% may undergo routine weekly nutritional assessments, enabling precision stratified care and optimizing the allocation of healthcare resources.

### 4.2 Risk factor analysis for malnutrition prediction models in lung cancer patients undergoing thoracoscopic resection

Preoperative ALB levels serve as a core indicator for assessing malnutrition risk and guiding nutritional interventions. The preoperative ALB levels <30 g/L, as designated by the Perioperative Nutrition Guidelines in the 2021 European Society for Parenteral and Enteral Nutrition (ESPEN) (28), is recommended as an indication for preoperative nutritional support. This metric has also been adopted as a key screening criterion by the Perioperative Nutrition Screen published by the Enhanced Recovery After Surgery Society (29), indicating the importance of preoperative ALB levels for effectively suggesting patient nutritional status. In this study, preoperative ALB level <35 g/L was an independent risk factor for postoperative malnutrition [OR = 0.90, 95% CI (0.80–1.00), P = 0.046], further confirming its clinical value in predicting outcomes for lung cancer patients after thoracoscopic resection. In some lung cancer patients, hypoalbuminemia has already existed preoperatively, resulting in further increased probability of developing postoperative hypoalbuminemia. The incidence of postoperative hypoalbuminemia has been reported to be ranged from 14% to 49.60% (30, 31). Consistent with previous data (32), this study recorded a preoperative incidence of 7.97% and a postoperative incidence as high as 61.40%. Critically, hypoalbuminemia can synergistically worsen respiratory dysfunction, given the already compromised postoperative pulmonary function in lung cancer patients, necessitating clinical emphasis on its prevention and management. Meanwhile, perioperative hypoalbuminemia has been recognized to be a risk factor for postoperative complications and even mortality (33). To certain extent, hypoalbuminemia can indicate potential malnutrition in some patients (34), possibly attributable to the activation of the hypothalamic-pituitary-adrenal axis by surgical stress that may induce a hypercatabolic state. Increased release of catabolic hormones can accelerate protein breakdown, while inflammatory cytokines (e.g., tumor necrosis factor, interleukin-6, etc.) may suppress ALB synthesis and disrupt the endothelial glycocalyx layer, thereby promoting vascular permeability and causing ALB leakage. Moreover, during the acute phase response, the liver prioritizes acute-phase protein synthesis over ALB production owing to preoperative gastrointestinal dysfunction and postoperative intake insufficiency. In addition, protein depletion may be further exacerbated given intraoperative blood loss and postoperative exudation. Collectively, these mechanisms may induce negative nitrogen balance, depletion of protein reserves, and deficiency of nutritional substrates. Concurrently, reduced plasma colloid osmotic pressure may trigger tissue edema, which may impair digestive absorption and metabolic efficiency, ultimately resulting in the presence of malnutrition.

Consistent with findings reported by Cruz-Jentoft et al. (35), advanced age is an independent risk factor for postoperative malnutrition in lung cancer patients following thoracoscopic surgery. Primarily, elderly patients experience gradual deterioration in health status and physical function, which may impair their food intake and nutrient absorption due to the resultant teeth loosening and loss, as well as weakened chewing, swallowing, and digestive function. As reported previously, only 9.1% of elderly patients met their daily energy requirements, while over half had energy intakes below 50% of estimated needs, and nearly 50% achieved only 75% of their protein requirements (36). Consequently, the risk of nutritional deficiency may be increased in the context of low nutrient intake, compounded by dietary monotony. Furthermore, these patients may have exacerbated cardiopulmonary burden given age-related changes in T-cell-mediated immune responses and inflammatory reactions. Meanwhile, the risk of malnutrition can be further increased considering an increased susceptibility to infections resulting from impaired airway mucosal function due to repeated inflammatory stimulation. Concurrently, socioeconomic factors (e.g., social isolation and limited income) may also contribute to heightened malnutrition risk in elderly patients (37). Collectively, all these interpretations underscore the clinical necessity to prioritize nutritional management for older patients with lung cancer, including developing personalized nutritional plans, ensuring adequate nutrient intake, and preventing dietary insufficiency-induced malnutrition.

The NRS2002 is an internationally recommended tool for nutritional screening. Beyond identifying patients with malnutrition or nutritional risk, preoperative nutritional screening can also predict clinical outcomes, thus benefiting preoperative nutritional therapy. Approximately 25% to 75% of cancer patients have been revealed to experience nutritional risk, with approximately 30% of deaths directly attributed to malnutrition (38–40). Here in our study, compared to the nutritional risk group, the non-nutritional risk group exhibited significantly shorter duration of postoperative thoracic drainage tube retention; and this group also had a notably reduced rate of postoperative complications and fewer patients needing adjuvant therapy. It may be explained by pre-existing nutritional deficiencies in lung cancer patients. Patients’ functional recovery may be impaired by factors such as major surgical trauma, significant intraoperative blood loss, postoperative wound pain, and surgical stress-induced high metabolic demands collectively. Moreover, patients experience shorter period of early postoperative ambulation, poor appetite, or anorexia, resulting in failure to meet their nutritional requirements. Concurrently, there is a delay of nutritional risk screening and intervention for lung cancer patients in China generally, leading to poor surgical tolerance. These factors, coupled with compromised immunity, may increase the risk of infection, severely impact clinical outcomes (41), and further exacerbate malnutrition. It is recommended that patients undergo NRS2002 nutritional screening upon admission. For patients with a score of ≥3 points, nutritional intervention should be initiated immediately by clinicians, such as prioritizing oral nutritional supplementation, supplemented with enteral or parenteral nutrition when necessary, and dynamically adjusting the intervention plan to reduce complications and improve prognosis.

ADL score is strongly linked to the incidence of malnutrition and is a significant determinant of nutritional status. Consistent with Duan et al. (42), this study observed a correlation of lower BADL scores with poorer nutritional status. Preoperative BADL scores serve as a vital indicator for assessing the functional capacity of patients. Patients with low scores may have diminished physiological reserve and functional status, resulting in reduced tolerance to surgery and recovery potential (43). Conversely, patients with high scores may experience reduced likelihood of malnutrition to some extent given their greater physical strength and mobility, as well as higher nutritional intake requirements and capacity. For instance, moderate physical activity has been discovered to enable the reduction of inflammation and promotion of beneficial nutritional metabolism (44). The ESPEN guidelines on nutrition and cancer (45) also incorporated physical activity into nutritional interventions. Meanwhile, patients with lung cancer insisting on exercise would have mitigated fatigue, enhanced quality of life, improved lung function, and boosted muscle mass (46). Therefore, perioperative BADL assessment should be implemented by clinical healthcare providers for elderly lung cancer patients, combined with guidance on appropriate exercise, and increase of daily activity levels to reduce the risk of malnutrition.

Furthermore, as a crucial therapeutic intervention for intrathoracic diseases, closed thoracic drainage inherently carries the risk of exacerbating malnutrition. Specifically, drainage may induce continuous loss of key nutrients (e.g., ALB, immunoglobulins, and electrolytes) from protein-rich pleural effusions, particularly bloody or exudative pleural effusions. Prolonged or excessive drainage can directly deplete the protein reserves in the body (47). Moreover, lung cancer resection may trigger chylothorax owing to the frequent damage to the thoracic duct. Chyle contains substantial ALB, and the loss of this protein, coupled with dietary restrictions [e.g., low-fat or medium-chain triglyceride (MCT) diets] implemented to reduce chyle secretion, may further exacerbate severe malnutrition and energy deficiency (48). Concurrently, patients’ appetite may be dampened by traumatic stress from tube placement, local pain stimuli, and mechanical irritation, accompanied by the inducement of gastrointestinal symptoms such as nausea and abdominal distension, and impaired capacity for nutrient digestion and absorption. Ultimately, these interconnected mechanisms drive the development of malnutrition.

Intraoperative blood loss is a significant contributor to malnutrition in lung cancer patients, with even higher risk in case of greater blood loss. In this type of surgery, major hemorrhage is common due to multiple factors as follows: **(1)** anatomical challenges (e.g., fragile hilar vascular walls, restricted surgical field, dense adhesions between pulmonary arteries and bronchi rendering lymph nodes susceptible to damage, and anatomical variations in pulmonary artery branches); and **(2)** surgical difficulties technically (e.g., difficult dissection of tumor-invaded vessels and technical challenges in instrument handling) (49). Owing to direct loss of plasma proteins, red blood cells, and trace elements, massive blood loss may cause hypoalbuminemia in those patients undergoing surgery. Concurrently, it may activate stress pathways and result in hypercatabolism. Patients may also develop impaired digestive and absorptive capacity given the presence of gastrointestinal mucosal ischemia and postoperative anemia. Blood transfusions and volume resuscitation may trigger dilutional hypoalbuminemia. In addition, as observed by Jiang Q et al. (50), decreased hemoglobin may induce systemic hypoxia and gastrointestinal hypoperfusion, further compromising the efficiency of nutrient absorption.

In addition, serum K is a significant predictor of postoperative nutritional deficiencies in lung cancer patients undergoing thoracoscopic surgery. serum K functions to regulate cellular metabolism, neuromuscular excitability, and gastrointestinal motility. Hypokalemia can cause muscle weakness, gastrointestinal smooth muscle paralysis (constipation and paralytic ileus), and reduced appetite, resulting in reduced activity levels in the affected patients (51). It can also suppress metabolic enzyme activity and exacerbate negative nitrogen balance, leading to impaired glycolysis and protein synthesis. Preoperatively, due to tumor catabolism, treatment-related side effects, and dietary restrictions, lung cancer patients often have inadequate potassium intake and depleted nutritional reserves. Intraoperative stress can also induce sympathetic activation and fluid loss, further disrupting potassium homeostasis. Patients following surgery may have gastrointestinal dysfunction, impaired energy metabolism, and complication-induced heightened metabolic demands, consequently leading to an exacerbation in the status of malnutrition. Clinically, there is a need to emphasize dynamic monitoring of serum K levels during the perioperative period. Simultaneous potassium supplementation and nutritional support are critical to break this vicious cycle and improve patient outcomes.

### 4.3 Clinical implications of the malnutrition risk predictive model for nursing practice in lung cancer patients undergoing thoracoscopic resection

According to the aforementioned findings, in the clinical practice of nursing, it inspire us to give priority to the monitoring of the following high-risk groups: elderly patients, those with hypoalbuminemia (ALB < 35 g/L), preoperative BADL scores < 90, preoperative NRS2002 scores ≥ 3, and patients with significant intraoperative blood loss or markedly increased postoperative drainage volume. This study further developed an online prediction model for risk assessment. Patients with a score > 40.68 would have high malnutrition risk, warranting immediate intervention; while patients with scores near this threshold can undergo more frequent dynamic screening based on the increased assessment frequency. In the future, complementary intervention measures will be developed and delivered to healthcare professionals and individual patients via digital platforms, with real-time monitoring of intervention outcomes.

Taking into consideration of findings in our study, it is recommended to carry out comprehensive perioperative nutritional interventions. Patients with preoperative ALB < 35 g/L should initiate oral nutritional supplementation one week preoperatively [e.g., 1.2–2.0 g/(kg·d) protein daily, administered in 2–3 divided doses]. Moreover, serum ALB levels should be monitored postoperatively; associated with the assessment of requirement for enteral-parenteral nutritional support by nurses in collaboration with physicians, if persistently below 30 g/L.

Simultaneously, preoperative rehabilitation plans should be developed by nurses for patients with preoperative BADL scores < 90, including: (1) aerobic exercise: brisk walking or cycling at Borg Scale intensity 13–16, 30–60 minutes per session, 3–5 times weekly to improve cardiopulmonary endurance; (2) resistance training: 6 exercises (e.g., seated knee raises, resistance knee extensions, chest presses), completing 8–12 repetitions per set, 2–3 sets per session, and 2–3 times weekly to strengthen limb muscles; (3) inspiratory muscle training: training by employing the “rapid inhalation, slow exhalation” technique with initial resistance set to 30%–50% of maximum inspiratory pressure (30 repetitions per set, and 1–2 sets daily), combined with the first two training components. Patients should be instructed to gradually enhance functional capacity and improve nutritional intake by using scientifically designed exercise regimens. Moreover, patients should be subjected to daily monitoring of drainage output (volume and characteristics), K, ALB, and hemoglobin concentrations for dynamic recovery assessment postoperatively. Cases with chylothorax cases should follow a low-fat/MCT diet; while cases with major hemorrhage should receive reinforced meticulous procedural care and hemostatic measures. Concurrently, there is a need to pay attention to the establishment of multidisciplinary intervention plan (52), including strengthening health education and psychological support for patients and families, creating malnutrition risk early warning systems, providing regular training for nursing staff, and integrating nutritional management strategies into routine care protocols. Altogether, multidimensional measures are necessitated for early prevention and intervention to reduce postoperative risks and enhance overall nursing quality.

## 5 Conclusion

This study develops a straightforward, user-friendly, and easily implementable predictive model, providing an effective tool for nursing staff to assess postoperative malnutrition in patients undergoing thoracoscopic resection for lung cancer. All indicators in this predictive model are easily accessible in clinical settings, thus demonstrating high practical utility. It is expected to benefit the screening of patients with high risk, thereby boosting the initiation of early interventions by nursing staff. In addition, the model also has validated applicability through external validation. Notably, the model’s predictive accuracy is reduced in high-nutritional-risk patients, requiring closer clinical monitoring.

## 6 Limitations

There are two major drawbacks to this study: (1) Model complexity and overfitting danger. Indeed, XGBoost model exhibits superior performance in processing complex data and non-linear interactions. However, additional model parameter tuning is still necessary to ensure the model’s stability and ability to generalize across different datasets. Future research may continue to improve the model and tools to better meet clinical practical needs, thereby expanding their application potential in clinical decision support. (2) Limited complication assessment. This study failed to measure the severity of complications by employing the Clavien-Dindo classification system. To increase predictive accuracy, the “complication severity grading” should be adopted as a model variable in our future research to examine the impact of various severity levels on the likelihood of malnutrition. (3) Performance variability in high-risk subgroups. While the model showed robust generalizability in the elderly and general population, its discriminative power was limited in the subgroup with high nutritional risk scores (NRS2002 ≥ 3) during external validation. This limitation may be attributed to the relatively small sample size of this subgroup and the high heterogeneity of patients with severe nutritional risk. Future studies should consider incorporating specific metabolic biomarkers to enhance prediction precision for this high-risk population. Furthermore, integrating dynamic postoperative changes (i.e., time-series data) will be a key priority in our future research to achieve continuous forecasting. (4) Unaddressed class imbalance. Postoperative malnutrition accounted for only 11.3% of total samples, leading to inherent class imbalance that was not addressed during data splitting. This may have biased model training toward the majority class and contributed to low Precision in validation sets, compromising predictive accuracy for clinically critical malnourished patients. Future studies will use class weight adjustment or Borderline-SMOTE to balance datasets and improve model robustness.

## Data Availability

The original contributions presented in the study are included in the article/**Supplementary Material**. Further inquiries can be directed to the corresponding author.

## Supplementary material

Supplementary Table Hyperparameter settings for the eight machine learning models.
