# Supplementary material for: Machine learning model for predicting malnutrition risk in lung cancer patients after thoracoscopic resection: a multi-center study
Source: Front Oncol. 2026 Feb 9;16:1727595. doi: 10.3389/fonc.2026.1727595 (PMC12926100; doi:10.3389/fonc.2026.1727595)
Supplement: Supplementary Table — Hyperparameter settings for the eight machine learning models. [file Table1.pdf]

Supplementary Table : Hyperparameter settings for the eight machine learning models

Model	Hyperparameters &Tuning Grid
Logistic Regression	Default parameters (glm)
SVM (Radial)	sigma = 0.001, C = 0.09
GBM	n.trees=100,interaction.depth=5, shrinkage=0.1, n.minobsinnode = 30
Neural Network	size=6, decay = 0.6
Random Forest	mtry = 11, numRandomCuts = 3
XGBoost	nrounds=200,max_depth=4,eta=0.05,gamma=0.3, colsample_bytree = 0.6, min_child_weight = 1, subsample = 0.7
KNN	kmax = 12, distance = 1, kernel = "optimal"
AdaBoost	mfinal = 2, maxdepth = 2, coeflearn = "Zhu"
